# Synovial Fluid and Serum Inflammation Biomarkers After Autologous Matrix-Induced Chondrogenesis (AMIC) for Knee Chondral Defects

**DOI:** 10.3390/jcm15051874

**Published:** 2026-02-28

**Authors:** Adrian Urbanek, Maciej Wrotniak, Zenon Czuba, Paweł Dolibog, Grzegorz Pilecki, Marcin Kostuj, Paulina Zalejska-Fiolka, Jolanta Zalejska-Fiolka

**Affiliations:** 1 Department of Biochemistry, Faculty of Medical Sciences in Zabrze, Medical University of Silesia, 40-055 Katowice, Poland; s91883@365.sum.edu.pl (P.Z.-F.); jzalejskafiolka@sum.edu.pl (J.Z.-F.); 2 Department of Medical Rescue, Faculty of Medical Sciences in Zabrze, Medical University of Silesia, 40-055 Katowice, Poland; 3 Department of Microbiology and Immunology, Faculty of Medical Sciences in Zabrze, Medical University of Silesia, 40-055 Katowice, Poland; zczuba@sum.edu.pl; 4 Department of Biophysics, Faculty of Medical Sciences in Zabrze, Medical University of Silesia, 40-055 Katowice, Poland; pawel.dolibog@sum.edu.pl; 5 Department of Pediatric Orthopedics and Traumatology, Municipal Hospital Complex, 41-500 Chorzów, Poland; 6 Department of Medical and Molecular Biology, Faculty of Medical Sciences in Zabrze, Medical University of Silesia, 40-055 Katowice, Poland; 7 Scientific Research Facility, Branch in Bielsko-Biała, Medical University of Silesia, 40-055 Katowice, Poland

**Keywords:** AMIC, MaioRegen, knee, cartilage defect, synovial fluid, IL-1β, IL-1RA, IL-6, inflammation, biomarkers

## Abstract

**Background:** Focal chondral and osteochondral knee defects have limited intrinsic healing capacity and may progress toward post-traumatic osteoarthritis. Early post-operative inflammatory signaling may influence clinical recovery after cartilage repair. This prospective, single-center observational cohort study aimed to characterize short-term post-operative inflammatory biomarker profiles in synovial fluid and serum after AMIC and to assess associations with patient-reported outcomes over 12 months. **Methods:** Fifteen patients undergoing autologous matrix-induced chondrogenesis (AMIC) for focal knee chondral/osteochondral defects were prospectively enrolled. International Knee Documentation Committee (IKDC) and Lysholm scores were recorded pre-operatively and at 6 and 12 months. Synovial fluid and serum were collected intraoperatively, at 6 and 12 weeks post-operatively. Interleukin (IL)-1β, IL-1 receptor antagonist (IL-1RA), and IL-6 were quantified using multiplex flow luminescence immunoassay, and the total synovial fluid protein level was measured. Non-parametric repeated-measures testing and Spearman’s rank correlation were applied (*p* < 0.05). **Results:** IKDC and Lysholm scores improved from (30.6 ± 9.4) to (58.8 ± 15.0) and from (57.5 ± 18.6) to (78.2 ± 14.7), respectively, exceeding established minimal clinically important difference (MCID) thresholds. Synovial fluid IL-1β and IL-1RA increased significantly over time ((*p* = 0.01357) and (*p* = 0.03953), respectively); IL-1β remained elevated, whereas IL-1RA tended to decline after 6 weeks. IL-6 levels remained low throughout. Total synovial fluid protein increased significantly (*p* = 0.00043). No significant correlations were observed between corresponding biomarker levels in synovial fluid and serum. Higher IL-6 and a higher IL-1β/IL-1RA ratio were associated with poorer clinical improvement (ρ = −0.80, *p* < 0.05 and ρ = −0.580, *p* < 0.05, respectively). **Conclusions:** AMIC was associated with a sustained intra-articular inflammatory response despite favorable 12-month outcomes. Exploratory analyses suggest that inflammatory dysregulation—particularly involving IL-6 and IL-1β/IL-1RA balance—may be linked to less favourable clinical recovery. Synovial fluid measurements provided more relevant information on local joint biology than serum sampling.

## 1. Introduction

Articular cartilage defects of the knee are a common condition that impairs patients’ quality of life. Because of the avascular and aneural nature of cartilage, focal defects have a limited capacity for intrinsic repair and may frequently lead to post-traumatic osteoarthritis (PTOA) [1,2]. Although the precise biochemical pathways underlying PTOA remain incompletely understood, it is well established that pro-inflammatory cytokines play a pivotal role in its pathogenesis [3]. Joint trauma and cartilage surgery result in rapid increases in pro-inflammatory cytokines such as interleukin (IL)-1β and IL-6 in synovial fluid and, to a lesser extent, in serum [4,5,6]. These lesions are frequently encountered in active adults and may initiate a synovial inflammatory cascade that can influence symptoms and repair tissue formation.

IL-1β is a major catabolic cytokine that stimulates the production of prostaglandins, nitric oxide, chemokines, and matrix-degrading enzymes like metalloproteinases and ADAMTS [7]. Its activity is balanced by the IL-1 receptor antagonist (IL-1RA), which binds IL-1 receptors and acts as a competitive inhibitor of IL-1 [7]. An imbalance of this axis, with relative predominance of IL-1β over IL-1RA, has been recognized in chronic synovitis and cartilage damage in rheumatoid arthritis and osteoarthritis [7,8].

IL-6 is produced by synoviocytes and chondrocytes in response to mechanical injury and cytokine stimulation, further linking inflammation to structural progression by promoting catabolic gene expression, subchondral bone remodeling, and pain [4,5].

In this context, biological repair techniques have been developed to delay or prevent degenerative changes in the knee joint. Autologous Matrix-Induced Chondrogenesis (AMIC) combines microfracture with a scaffold to stabilize the marrow clot and support the formation of repair tissue. In the present study, AMIC was performed using a collagen–hydroxyapatite scaffold (MaioRegen^®^, Finceramica S.p.A., Faenza, Italy); published clinical series have reported post-operative improvement following this approach in symptomatic focal knee defects [9,10].

Several clinical studies have examined synovial and serum cytokines after various injuries and procedures, such as anterior cruciate ligament (ACL) reconstruction and meniscectomy, demonstrating marked, time-dependent changes in IL-1β, IL-1RA, and IL-6, and suggesting that specific cytokine profiles may contribute to, or protect from, subsequent joint degeneration [4,5,11]. However, to date, the early post-operative dynamics of inflammatory processes after AMIC have not been described. In addition to marrow stimulation, a collagen–hydroxyapatite membrane is implanted into the defect, introducing a foreign material into the joint and potentially modifying the local inflammatory response.

The aim of the present prospective study was therefore to characterize the short-term post-operative profile of inflammatory cytokines in synovial fluid and serum in patients undergoing AMIC with a MaioRegen^®^ scaffold for focal chondral or osteochondral defects of the knee, and to assess their association with clinical outcomes over a 12-month follow-up period. We hypothesized that AMIC would induce a transient intra-articular inflammatory response, that an unfavorable balance between IL-1β and IL-1RA and increased IL-6 activity would be associated with poorer improvement in patient-reported outcome measures, and that synovial cytokine levels would correlate only weakly with corresponding serum measurements. Given the study design and sample size, analyses of biomarker–outcome associations were intended as exploratory and hypothesis-generating.

## 2. Materials and Methods

### 2.1. Subjects

This prospective, single-center observational cohort study was conducted at a regional orthopedic hospital in Sosnowiec, Poland. The fifteen patients undergoing surgical treatment for focal, symptomatic cartilage lesions of the knee were prospectively enrolled in this study. Consecutive eligible patients scheduled for AMIC between July 2023 and November 2024 were invited to participate. The mean age was 44.1 ± 9.1 years, mean BMI 29.1 ± 3.4 kg/m^2^, and mean defect size 2.3 ± 0.9 cm^2^. Inclusion criteria were isolated, focal, symptomatic chondral lesions of the knee, the ability to comply with follow-up visits, and the sampling schedule. Exclusion criteria included: radiographic or clinical osteoarthritis, age > 60 years, mechanical axis malalignment, ligamentous injuries resulting in instability, clinically relevant meniscal tears, inflammatory or rheumatologic diseases, intra-articular injection in the 3 months before surgery, prior knee surgery, and knee surgery within the follow-up period. Lesions were classified as ICRS grade III–IV on pre-operative MRI and confirmed intraoperatively. (27 + 30) Defect location included the medial femoral condyle (*n* = 12), patella (*n* = 2), and combined patella + medial femoral condyle (*n* = 1); etiology was categorized clinically as traumatic or degenerative. (30) AMIC was selected as a single-stage cartilage repair option for this cohort; compared with osteochondral autograft transfer (OATS), it avoids donor-site morbidity and graft availability limitations while providing scaffold stabilization beyond microfracture alone. All participants provided written informed consent for the use of biological material collected during surgery. The study protocol was approved by the local Ethics Committee.

### 2.2. Surgical Technique

Diagnostic arthroscopy was performed initially to confirm the pre-operative diagnosis and cartilage lesion grade. All AMIC procedures were performed through a mini-arthrotomy. After exposure, the edges of the lesion were prepared, and sclerotic bone was removed. Micro-drilling was then performed, and the membrane was press-fitted into the defect and secured with fibrin glue. During the same procedure, limited partial medial meniscectomy was performed when required (*n* = 7), and in one patient, AMIC was augmented with autologous bone grafting from tibial epiphysis. Post-operative rehabilitation followed a structured protocol with restricted weight-bearing with crutches for the first 6 weeks, early range-of-motion exercises as tolerated, staged strengthening and proprioceptive training thereafter, and return to running/sport only after functional recovery and clinical approval.

### 2.3. Outcomes of Interest

Patients were evaluated pre-operatively and prospectively by the International Knee Documentation Committee (IKDC) score and Lysholm functional scale before the surgery and at 6 and 12 months of follow-up. Questionnaires were filled out by patients, with minimal assistance when needed.

Synovial fluid was aspirated aseptically using a sterile needle and syringe, without lavage, at three time points: intraoperatively, immediately before the AMIC procedure, and at six and twelve weeks post-operatively. Immediately after collection, samples were centrifuged by trained medical personnel to remove cellular debris. A sufficient volume for biochemical analysis was obtained; exact aspirated volumes were not systematically recorded, and no dilution procedures were used. In some cases, mild hemarthrosis or blood contamination was observed, but centrifugation allowed separation of a clear supernatant for analysis. At the same time points, venous blood samples were collected for serum analysis. Biological samples were transported on dry ice and stored at −83 °C until biochemical analysis. Samples were not thawed prior to analysis, and all assays were performed simultaneously in a single analytical run. Concentrations of IL-1β, IL-1RA, and IL-6 were quantified using a multiplex Flow Luminescence Immunoassay (FLIA) according to the manufacturer’s instructions (Cloud-Clone Corp., Katy, TX, USA). Total protein concentration in synovial fluid samples was determined using the biuret method, according to standard laboratory procedures.

### 2.4. Sample Size

On the basis of the results of the pilot study, we estimated the sample size necessary to demonstrate a statistical significance of *p* = 0.05 and a test power of 0.8 within the group scores. The resulting minimum sample size was 14 patients in the study group. The target sample size was 15. The sample size analysis was performed using STATISTICA v. 13.3 (TIBCO Software Inc., Palo Alto, CA, USA).

### 2.5. Statistical Analysis

Statistical analyses were performed using STATISTICA v. 13.3 (TIBCO Software Inc., Palo Alto, CA, USA) and PQStat v. 1.8.4 (PQStat Software, Poznań, Poland). Data distribution was evaluated using the Shapiro–Wilk test. Because most variables were non-normally distributed, non-parametric tests were applied. Percentage change in measured values (Lysholm knee score, IKDC score, and biomarkers) was calculated using the following formula: (Final value − Initial value)/Initial value × 100%. To compare values obtained pre-operatively and at two post-operative time points, the non-parametric Friedman Anova test and post hoc Dunn–Bonferroni test for repeated measures were used. Correlations between biochemical markers and clinical outcomes were assessed using Spearman’s rank correlation coefficient. A *p*-value < 0.05 was considered statistically significant.

## 3. Results

### 3.1. Clinical Outcomes

Clinical outcomes were assessed by changes in PROMs between the pre-operative assessment and the 6 and 12-month follow-up. Mean pre-operative International Knee Documentation Committee (IKDC) score was 30.6 ± 9.4, which improved to 51.8 ± 15.2 at 6 months and 58.8 ± 15.0 at 12 months post-operatively. Mean Lysholm knee score increased from a pre-operative value of 57.5 ± 18.6 to 70.3 ± 15.9 at 6 months and 78.2 ± 14.7 at the 12-month follow-up. Data are presented in Table 1 and Table 2. For both outcome measures, the observed improvements exceeded the established minimal clinically important difference (MCID) thresholds (IKDC = 16.7; Lysholm = 10.1), which have been previously applied in cartilage repair outcome studies [12]. MCID was assessed using group mean change; individually, two patients did not reach MCID thresholds despite overall improvement at the group level.

### 3.2. Biomarker Levels in Synovial Fluid

**Figure 1:**
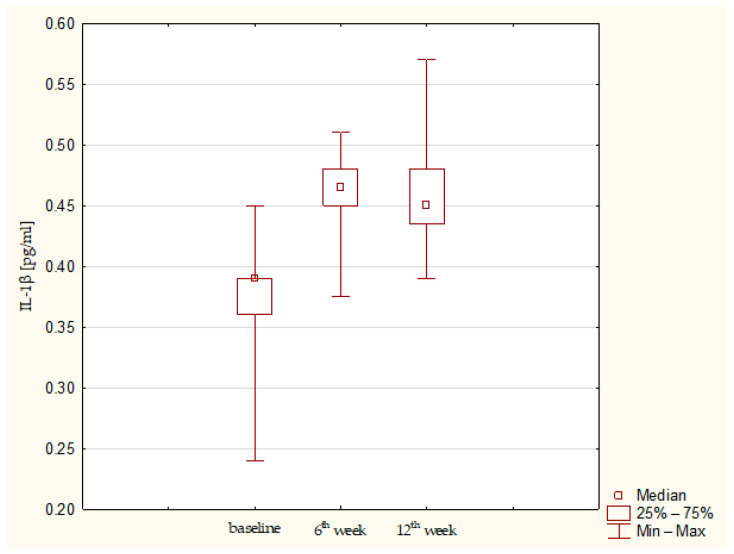
IL-1β concentration in synovial fluid at baseline, 6th week, and 12th week. Abbreviations: 25%—first quartile; 75%—third quartile; min—minimum value; max—maximum value.

**Figure 2:**
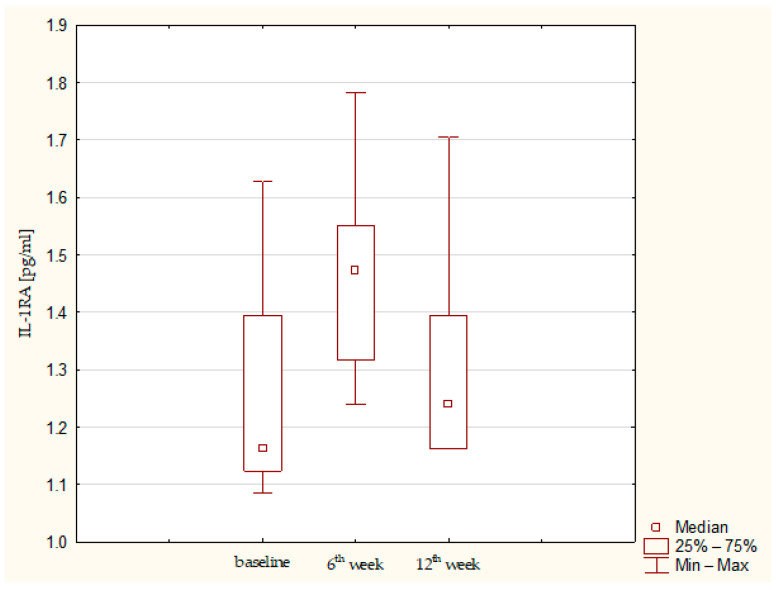
IL-1RA concentration in synovial fluid at baseline, 6th week, and 12th week. Abbreviations: 25%—first quartile; 75%—third quartile; min—minimum value; max—maximum value.

**Figure 3:**
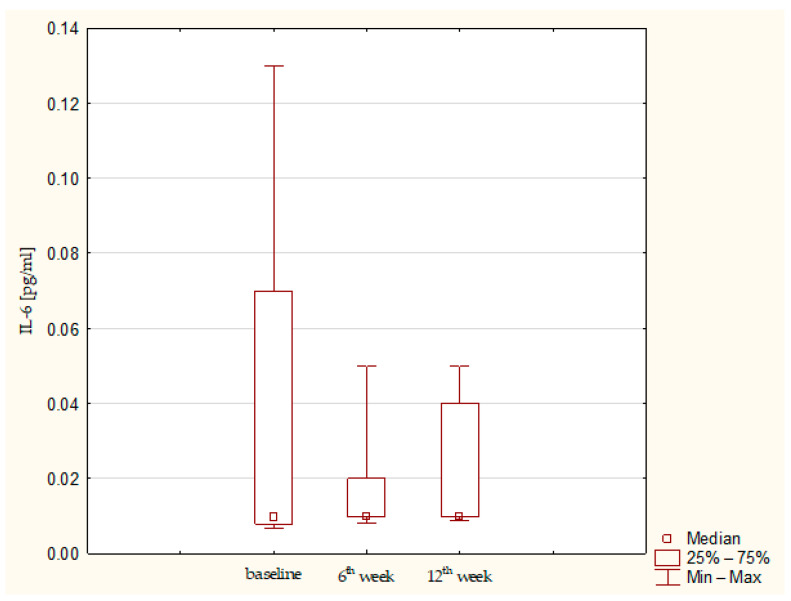
IL-6 concentration in synovial fluid at baseline, 6th week, and 12th week. Abbreviations: 25%—first quartile; 75%—third quartile; min—minimum value; max—maximum value.

**Figure 4:**
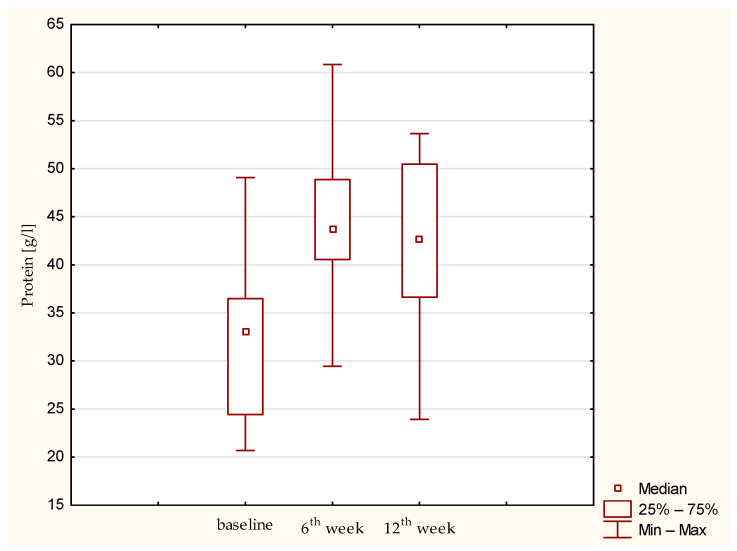
Protein concentration in synovial fluid at baseline, 6th week, and 12th week. Abbreviations: 25%—first quartile; 75%—third quartile; min—minimum value; max—maximum value.

Synovial fluid and serum samples from fifteen patients were analyzed. A statistically significant increase in IL-1β (*p* = 0.01357) and IL-1RA (*p*= 0.03953) was observed. IL-1β remained elevated throughout the observation period, whereas IL-1RA showed a tendency to decrease after the sixth post-operative week (Figure 1 and Figure 2). IL-6 concentrations were low at baseline and remained low up to the 12th week (Figure 3). Total protein levels followed a pattern similar to IL-1β, showing a significant increase and remaining stable thereafter (*p* = 0.00043) (Figure 4). Concurrent elevation of IL-1β, IL-1RA, and protein indicates the presence of a sustained intra-articular inflammatory response after AMIC, consistent with observations reported for other arthroscopic knee procedures. Detailed results are presented in Table 3.

### 3.3. Comparison with Normal Levels Reported in Literature

When compared with available literature, the absolute concentrations of cytokines observed in our study were generally low (Table 4). IL-1β was markedly elevated, almost 3-fold higher than the normal level, whereas IL-1RA concentrations were close to values reported under normal conditions, and IL-6 levels were unexpectedly low. Reference values were taken from the study by Iversen et al., in which synovial fluid cytokine concentrations were compared between fractured knees and the contralateral knees considered clinically healthy [13]. Total protein concentrations in synovial fluid were close to reported normal values at baseline (31.7 g/L vs. approximately 30 g/L) and increased to levels typical of inflammatory synovial fluid (>25 g/L; 44.8 g/L and 43.8 g/L at 6 and 12 weeks, respectively) [14]. This supports adequate sample quality and argues against major technical or pre-analytical errors.

### 3.4. Correlations Between Cytokine Levels in Synovial Fluid and Serum

There was no significant correlation between the assessed cytokine levels in synovial fluid and serum. IL-1β exhibited a similar pattern in both compartments—an increase up to 6 weeks followed by a decline between 6 and 12 weeks—although these changes did not reach statistical significance. While no significant correlations were observed for individual biomarkers between compartments, a strong positive correlation was found between IL-1β levels in synovial fluid and IL-1RA in serum at 12 weeks (ρ = 0.7778, *p* < 0.05). This association is consistent with the biological role of IL-1RA as a secondary response following IL-1β–driven inflammation and suggests a limited, indirect linkage between local and systemic inflammatory signaling. As serum biomarker levels generally failed to reflect the local knee environment, they were not further related to clinical outcomes. Data on blood cytokine concentrations are presented in Appendix A.

### 3.5. Correlations Between Cytokine Levels and Clinical Outcomes

IL-1β, a key pro-inflammatory cytokine involved in intra-articular inflammatory signaling, showed only weak to moderate correlations with clinical outcomes and did not reach statistical significance. In contrast, a significantly strong to very strong inverse correlation was observed between IL-1RA levels at 12 weeks and the Lysholm score (ρ = −0.764, *p* = 0.045). This finding may suggest that higher IL-1RA levels primarily reflect a heightened inflammatory state within the joint, rather than indicating an anti-inflammatory effect of this cytokine. Given the observational design, small cohort, and multiple correlation analyses, biomarker–outcome associations should be regarded as exploratory and interpreted cautiously, particularly when considering clinical implications.

**Figure 5:**
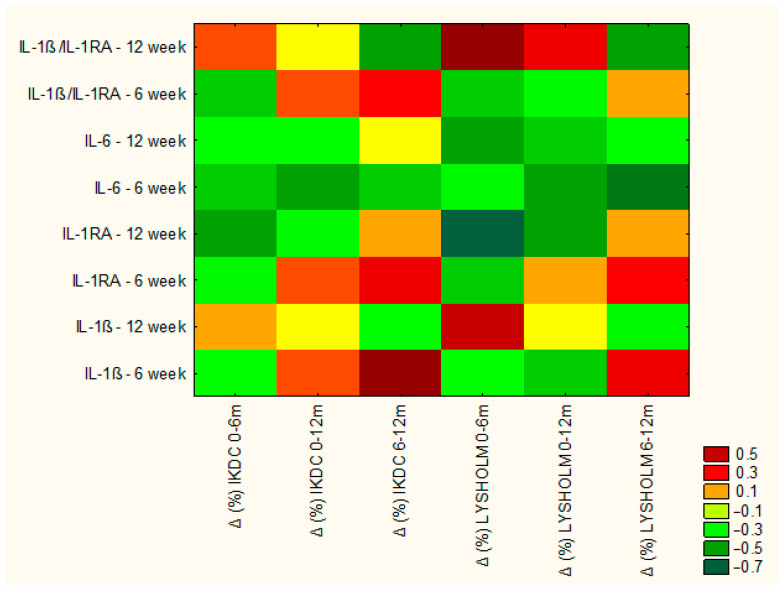
Heatmap illustrating Spearman’s rank correlations (ρ) between cytokine concentrations (pg/mL), the IL-1β/IL-1RA ratio, and changes (Δ) in clinical scores. Biomarker concentrations were measured at 6 and 12 weeks post-operatively. Percentage changes in clinical scores were calculated for 0–6 months (baseline to 6 months), 0–12 months (baseline to 12 months), and 6–12 months post-operatively. Color intensity represents the strength of the correlation, with warmer colors indicating positive correlations and cooler colors indicating negative correlations.

A significant, very strong negative correlation was also identified between IL-6 concentrations in the 6th week and surgical outcome (ρ = −0.80, *p* = 0.016), supporting the hypothesis that a sustained inflammatory response following the AMIC procedure may contribute to less favorable clinical recovery in some patients. The overall pattern of cytokine–outcome associations is illustrated in the correlation heatmap (Figure 5).

To further characterize the balance between pro- and anti-inflammatory signaling, the IL-1β/IL-1RA ratio was analyzed. It provides a more integrative measure of the intra-articular inflammatory state than assessment of either cytokine alone and has been reported to be elevated in persistent joint inflammation. Consistent with this concept, the IL-1β/IL-1RA ratio showed a significant negative correlation with mid-term improvement in IKDC score (ρ = −0.580, *p* = 0.028), suggesting that a relative dominance of catabolic over anti-inflammatory processes may adversely affect knee function and early cartilage repair following AMIC. These associations are summarized in Figure 5.

## 4. Discussion

To our knowledge, this is the first study to evaluate the profile of intra-articular inflammatory biomarkers in patients undergoing AMIC. As expected, an inflammatory response within the knee joint was observed, peaking at 6 weeks and remaining elevated at 12 weeks post-operatively. Overall, patient-reported outcomes at 12 months were favorable and exceeded the minimal clinically important difference; however, persistent inflammatory signaling, reflected by IL-6 levels and the IL-1β/IL-1RA balance, was associated with poorer clinical outcomes. These observations should be interpreted with considerable caution, given the small sample size and the absence of a surgical comparator cohort, as the observed inflammatory response cannot be clearly attributed to collagen scaffold implantation alone but may also reflect the effects of arthrotomy and defect preparation.

Schmal et al. [15] is one of the few studies assessing the intra-articular environment after surgical focal chondral defect repair. They studied patients who underwent microfracture and autologous chondrocyte implantation. IL-1β concentration was measured on 0, 1, and 2 days after surgery, and demonstrated a marked post-operative increase and a decrease the next day. However, the material was collected from post-operative drainage, which is typically contaminated with blood and may bias results [15]. Another study, regarding synovial fluid biomarkers after ACI, did not test inflammatory markers but evaluated degradation and cartilage formation markers [16].

In osteoarthritis, inflammatory processes play a well-established role in disease progression and may serve as a biological reference for the present study, representing an end-stage of cartilage damage. After HTO, a significant post-operative reduction in synovial fluid IL-6, MMP-3, and COMP concentrations was observed, while IL-1β levels remained unchanged. Despite significant clinical improvement, alterations in inflammatory cytokines and matrix degradation markers did not correlate with cartilage status in arthroscopy [17]. After PRP and HA injections in osteoarthritis, synovial fluid concentrations of IL-1β, IL-1RA, and IL-6 were low, with IL-1β levels at 12 weeks, 0.14 and 0.34 pg/mL, respectively, and no significant changes were observed for IL-6 or IL-1RA. Correlations between cytokine levels and clinical outcomes were not statistically significant [18].

Dynamics of intra-articular inflammatory processes are well documented based on ACL-related injuries. In Catterall et al., inflammatory biomarkers in synovial fluid showed higher values early (approximately 2–3 weeks) after injury, with a tendency to decrease at the 6–8-week follow-up [4]. In a time interval between 1 and about 7 weeks after injury, Kingery et al. reported a significant drop in IL-6 and IL-1RA [19]. Similarly, Bigoni et al. reported elevated IL-1β and IL-6 in the acute phase after ACL rupture, but with IL-1β returning toward “normal” levels in the earlier, subacute window (3–15 days), based on cross-sectional time-group comparisons [5]. These findings altogether suggest the presence of a high initial inflammatory response with a tendency toward rapid resolution. The more sustained inflammatory profile observed in our cohort may reflect the combined effects of mini-arthrotomy, lesion-site preparation, and collagen membrane implantation, which could prolong intra-articular inflammatory signaling compared with injuries involving ligaments/menisci. However, because sampling was not performed in the first post-operative days, early inflammatory peaks may have been missed, which limits direct comparisons with studies focused on the acute phase, particularly as the ACL studies cited above capture biomarker dynamics within the first days to weeks after injury, whereas our first post-operative assessment was performed at 6 weeks.

With the exception of the linkage between synovial fluid IL-1β and serum IL-1RA, no significant correlations were observed between biomarker levels in synovial fluid and serum, indicating that local joint biological activity may not be reliably assessed with blood sampling. Catterall et al. [4] evaluated correlations between 12 biomarkers measured in paired synovial fluid and serum samples and found significant associations only for a subset of markers, while IL-1β—consistent with our findings—showed no significant synovial fluid–serum correlation. In another study following ACL reconstruction, serum IL-1β was detected only in cases with high synovial fluid IL-1β levels; however, this was an indirect comparison and was not supported by formal correlation analysis, making the findings uncertain [20]. A single study in patients with end-stage knee osteoarthritis reported a weak, but statistically significant correlation between synovial fluid and serum IL-6 levels [21].

Taken together, the present findings indicate that AMIC is associated with a measurable and temporally sustained intra-articular inflammatory response, which appears to be only partially comparable to that observed after ligamentous injury or other cartilage-related interventions. While synovial fluid IL-1β concentrations were elevated, IL-1RA and IL-6 levels remained low in absolute terms. The unexpectedly low absolute IL-6 concentrations may reflect a combination of biological factors and methodological influences (including assay sensitivity at low ranges and pre-analytical variability), which limits direct comparison of absolute IL-6 values across studies. An increase in IL-1RA may also reflect a compensatory, but insufficient, anti-inflammatory response rather than a direct indicator of adverse biological activity. Specific inflammatory signaling patterns—particularly involving IL-6 and the IL-1β/IL-1RA balance—were associated with less favorable clinical outcomes, underscoring the potential functional relevance of inflammatory dysregulation. Collectively, these observations may suggest that inflammatory processes play a relevant role in the biological response following AMIC and are more appropriately assessed through synovial fluid analysis rather than systemic blood measurements.

## 5. Limitations

This study has several limitations. First, the small sample size (*n* = 15) limits statistical power and generalizability, particularly for correlation analyses.

Procedural heterogeneity (concomitant partial meniscectomy in seven patients and bone graft augmentation in one case) may have influenced intra-articular inflammation; subgroup or sensitivity analyses were not feasible due to the sample size. In addition, synovial fluid and serum were collected intraoperatively and at 6 and 12 weeks post-operatively; early post-operative inflammatory peaks within the first days after surgery may have been missed.

Exact synovial fluid volumes were not systematically recorded, and occasional hemarthrosis was observed; although samples were centrifuged to obtain a clear supernatant, variability in blood contamination or dilution effects may have influenced measured biomarker concentrations and should be considered when interpreting absolute values.

Clinical outcomes were assessed using PROMs only; no imaging (MRI) or second-look arthroscopy was included to correlate biomarker profiles with structural cartilage repair. Therefore, biomarker–outcome associations cannot be directly interpreted as indicators of structural cartilage repair quality.

A limitation of this study is the lack of a control group. There are two main reasons for this. The amount of synovial fluid in a healthy knee is minimal, and saline dilution is often required for aspiration, which may cause pre-analytical errors. Secondly, collecting synovial fluid from healthy controls at three time points raises ethical concerns. For these reasons, our results were compared with published data from “healthy” control groups. Alternative surgical comparator cohorts (e.g., microfracture alone or other cartilage repair techniques) were not included, as this would require a different study design and indication pathway and was outside the scope of this descriptive biomarker study.

The observed cytokine concentrations were generally low but comparable to reported reference values, except for IL-6, which was markedly lower. The reason for that remains unclear, as the presence of intra-articular inflammation was supported by increased IL-1β, subsequent elevation of IL-1RA, and increased total protein levels. Very low IL-6 concentrations may also reflect methodological constraints, including values near assay detection limits and sensitivity to pre-analytical handling, which limits between-study comparison of absolute IL-6 levels. Here, it should be emphasized that reported synovial fluid cytokine concentrations vary substantially across studies and may differ by several-fold. For example, reported “normal” values range from 10.4 pg/mL for IL-1β [20], 241 pg/mL for IL-1RA [22], and 64 ± 120 pg/mL for IL-6 [23]. These differences may arise from biological heterogeneity or methodological differences, like dilution, sample handling, and the assay used. Therefore, synovial fluid cytokine measurements appear particularly valuable for within-cohort comparisons, provided that all samples are collected, processed, and analyzed using identical protocols.

## 6. Conclusions

AMIC cartilage repair was associated with a sustained intra-articular inflammatory response detectable up to 12 weeks postoperatively. Despite favorable clinical improvement at 12 months, specific inflammatory signaling patterns—particularly involving IL-6 and the IL-1β/IL-1RA balance—were associated with less favorable functional recovery. These findings support the concept that subtle inflammatory dysregulation may influence short- to mid-term clinical outcomes after AMIC and highlight the value of synovial fluid analysis as a more informative tool for assessing intra-articular biology than systemic serum measurements. However, given the exploratory design, limited sample size, and absence of a surgical comparator cohort, the results should be interpreted with caution.

## Figures and Tables

**Table 1:** Lysholm knee score. Abbreviations: ± SD (Standard deviation).

Timepoint	Value (Mean)	±SD
Pre-operative	57.5	18.6
6 months after surgery	70.3	15.9
12 months after surgery	78.2	14.7

**Table 2:** IKDC score. Abbreviations: ± SD (Standard deviation).

Timepoint	Value (Mean)	±SD
Pre-operative	30.6	9.4
6 months after surgery	51.8	15.2
12 months after surgery	58.8	15.0

**Table 3:** Biomarker concentrations in synovial fluid. Abbreviations: ± SD (Standard deviation).

Biomarker	Timepoint	Concentration (Mean)	±SD	Median	Minimum	Maximum
IL-1β (pg/mL)	Pre-operative	0.3733	0.0602	0.3900	0.2400	0.4500
	6th week post-op	0.4617	0.0388	0.4650	0.3750	0.5100
	12th week post-op	0.4617	0.0570	0.4500	0.3900	0.5700
IL-1RA (pg/mL)	Pre-operative	1.2455	0.1956	1.1625	1.0850	1.6275
	6th week post-op	1.4614	0.1758	1.4725	1.2400	1.7825
	12th week post-op	1.3120	0.1904	1.2400	1.1625	1.7050
IL-6 (pg/mL)	Pre-operative	0.0307	0.0411	0.0096	0.0068	0.1300
	6th week post-op	0.0160	0.0131	0.0100	0.0082	0.0500
	12th week post-op	0.0207	0.0181	0.0098	0.0089	0.0500
Protein total (g/L)	Pre-operative	31.7060	8.2275	32.9883	20.6852	49.0926
	6th week post-op	44.8341	8.7489	43.7376	29.4526	60.8477
	12th week post-op	42.7972	8.5670	42.6206	23.9254	53.6568

**Table 4:** Peak cytokine concentrations in SF from knees after AMIC procedure, compared to normal levels, reported in the literature [13].

Cytokine	Concentrations After AMIC (Mean)	Normal Concentrations from Literature (Mean)
IL-1β (pg/mL)	0.4617	0.127
IL-1RA (pg/mL)	1.4614	1.85
IL-6 (pg/mL)	0.0207	0.216

## Data Availability

The data presented in this study are available on reasonable request from the corresponding author. The data are not publicly available due to privacy and ethical restrictions related to patient confidentiality.

## Abbreviations

The following abbreviations are used in this manuscript:

AMIC	Autologous matrix-induced chondrogenesis
OA	Osteoarthritis
SF	Synovial fluid
HTO	High tibial osteotomy

## Appendix A

**Table A1:** Concentrations of IL-1β, IL-1RA, IL-6 in serum.

Biomarker	Timepoint	Concentration (pg/mL, g/L for Protein)	Std. Deviation	Median	Minimum	Maximum
IL-1β	Pre-operative	0.5904	0.4718	0.4350	0.3300	2.1100
	6th week post-op	0.52	0.1268	0.5025	0.4050	0.9000
	12th week post-op	0.4488	0.0413	0.4500	0.3750	0.5100
IL-1RA	Pre-operative	1.4190	0.3057	1.3175	1.1238	2.1716
	6th week post-op	1.6372	0.4582	1.4725	1.1625	2.7900
	12th week post-op	1.3950	0.1509	1.3563	1.2400	1.7825
IL-6	Pre-operative	0.0309	0.0397	0.0096	0.0086	0.1300
	6th week post-op	0.0691	0.0838	0.0500	0.0093	0.3300
	12th week post-op	0.0326	0.0408	0.0098	0.0082	0.1100
